# Preparation of Oxidized Starch/β-Lactoglobulin Complex Particles Using Microfluidic Chip for the Stabilization of Astaxanthin Emulsion

**DOI:** 10.3390/foods11193078

**Published:** 2022-10-04

**Authors:** Tianxing Wang, Lulu Zhang, Ling Chen, Xiaoxi Li

**Affiliations:** Ministry of Education Engineering Research Center of Starch and Protein Processing, Guangdong Province Key Laboratory for Green Processing of Natural Products and Product Safety, School of Food Science and Engineering, South China University of Technology, Guangzhou 510640, China

**Keywords:** microfluidics, oxidized starch, β-lactoglobulin, complex coacervation, emulsion stability

## Abstract

Here, we designed an oxidized starch/β-lactoglobulin (OS/β-lg) complex colloidal particle using a dual-channel microfluidic chip for the stabilization of astaxanthin emulsion. The effect of the mixing ratio, pH, and the degree of substitution (DS) of the oxidized starch on the formation of OS/β-lg complex particles was investigated in detail. The optimal complexation occurred at a pH of 3.6, a mixing ratio of 2:10, and a DS of 0.72%, giving an ideal colloidal particle with near-neutral wettability. With this optimum agent, the astaxanthin-loaded oil-in-water emulsions were successfully prepared. The obtained emulsions showed the typical non-Newton fluid behavior, and the rheological data met the Herschel–Bulkley model. The microscopic images confirmed the dense adsorption of the particle on the oil/water interface. In vitro release and stability studies demonstrated this compact layer contributed to the controlled-release and excellent stability of astaxanthin emulsions facing heat, ultraviolet, and oxidative intervention. This work suggests the potential of microfluidics for the production of food-grade solid emulsifiers.

## 1. Introduction

The marine-sourced astaxanthin (AST), a wine-colored carotenoid, presents greater antioxidant ability due to the presence of hydroxyl and ketonic end groups in each ionone ring [1]. The persistent consumption of AST can endow many benefits to human health including the prevention of inflammation, cancer, diabetes, and ageing [2,3]. However, its application was limited due to poor solubility, low bioavailability, and easy degradation in the face of adverse conditions [4]. Recently, Pickering emulsions have been deeply developed to improve the survival and bioavailability of AST [5]. For this type of emulsion, solid emulsifiers are the key point to resolve the stability and safety issues of food-grade emulsions [6]. In this respect, a large number of studies have reported the potential of biopolymer-based particles for emulsion stabilization, ranging from cellulose, dextrin, chitosan, protein, phytosterol to starch [7,8,9].

Among them, protein particles have been widely used as emulsifiers to stabilize Pickering emulsions due to their amphiphilicity. For instance, when heated over a denaturation temperature, β-lactoglobulin (β-lg) aggregates can be formed through hydrophobic and covalent interactions to prepare Pickering emulsions [10]. However, due to their preferred hydrophobicity, β-lg particle-stabilized emulsions are susceptible to coalescence once exposed to adverse environment factors, resulting in it being hard to prepare long-term stable Pickering emulsions using individual β-lg nanoparticles [11]. The application of protein/polysaccharides complex coacervation is the most reported approach to regulate the surface wettability of protein-rich colloidal particles [12]. Oxidized starch (OS) is an amphiphilic polysaccharide with unique features such as biocompatibility and low viscosity, which can be constantly used as food additives in various food matrix environments [13]. The literature reported many examples of protein/polysaccharide complex coacervation, such as β-lg/acacia gum, pea protein isolate/gum Arabic, and gelatin/pectin, to deliver oil-soluble ingredients such as astaxanthin. Therefore, it is theoretically feasible to accommodate the emulsifying properties of β-lg nanoparticles by incorporating OS into β-lg particles.

On the other hand, the prepared protein/insoluble-polysaccharide complex particle, through the direct mixing of two oppositely charged biopolymer dispersions and phase separation, normally gives a microscale particle size and polydispersity structure, restricting the event adsorption of solid particles at the oil–water interface of the emulsion droplets. Therefore, it is urgent to seek new approaches or techniques for the preparation of complex particles with a well-defined monodispersed and homogeneous structure in terms of shape and size. Microfluidics involves the precise handling of a small volume of fluids to fabricate a large number of microparticles in microchannels [14], with a number of advantages including the laminar flow of the fluids, the short diffusion distance of the molecules in the different phases, a high degree of control over the fluid dynamics, continuous flow operations, and low mixing time and power consumption [15]. Valencia et al. reported the preparation of core–shell lipid polymeric nanoparticles using microfluidics in a single-step nanoprecipitation process [16]. This makes it is workable for the controllable fabrication of complex particles inside a glass capillary with uniform size, narrow size distribution, and the desired composition [15]. In this case, microfluidics is considered as an alternative technique to incorporate the emulsifying, swelling, and gelling properties of OS and β-lg molecules to obtain complex colloidal particles with a well-defined emulsifying performance. To the best of our knowledge, little information is available on the formation of OS/β-lg complex particles through microfluidic chips, and no systematic study exists on OS/β-lg complex particles as Pickering emulsion emulsifiers.

Hence, this work prepared a well-designed OS/β-lg complex colloidal particle using a dual-channel microfluidic chip. We investigated the effect of the pH, mixing ratio, and the degree of carboxyl substitution (DS) on OS/β-lg complex coacervation through the measurement of particle size, ζ-potential, turbidity, critical pH values, and the contact angle of the obtained particles. Then, we selected the complex particles prepared at a pH of 3.6, a mixing ratio of 2:10, and DS of 0.72% to stabilize the AST emulsions and conducted the droplet size, optical image, confocal laser scanning microscopy (CLSM), and rheological measurements. Moreover, the storage stability of AST-loaded emulsions facing heat, ultraviolet, and oxidative intervention were evaluated. In addition, the release behavior of AST-loaded emulsions and their bioavailability were also discussed. The present study may provide references to design protein/polysaccharide complex particles using microfluidics for the stabilization of Pickering emulsions.

## 2. Materials and Methods

### 2.1. Materials

Oxidized corn starch was synthesized according to the literature methods with slight modifications [17]. See Table S1 and Figure S1 for more structural, detailed information. β-lactoglobulin, pancreatin, glucosidase, and pepsin were purchased from Sigma-Aldrich Co., Ltd. (St. Louis, MO, USA). Astaxanthin, bile salt, and α-amylase were purchased from Shanghai Macklin Biochemical Co., Ltd. (Shanghai, China). Medium-chain triglycerides (MCT) were purchased from Shanghai Source Leaf Biological Technology Co., Ltd. Sodium hypochlorite, methanol, and all other analytical grade reagents were purchased from Sinopharm Chemical Reagent Company (Shanghai, China). Microfluidic chip (68 mm × 95 mm × 4 mm, liquid holdup: 0.51 mL) and two-channel syringe pump system were purchased from Suzhou Wenhao Microfluidic Technology Co., Ltd., Suzhou, China.

### 2.2. Stock Solution Preparation

The β-lg and OS stock solutions (1.0, 1.5, 2.0, 3.0% *w*/*v*) were prepared by dissolving the β-lg and OS powder in deionized water. The stock solutions were constantly stirred at room temperature for 24 h to give a complete hydration.

### 2.3. Preparation of OS/β-lg Complex Coacervates through Microfluidic Platform

The OS stock solutions (OS-0.25, OS-0.72, OS-1.65) and β-lg stock solution were individually aspirated into two-channel syringe pump. Then, both were injected into the microfluidic chip to obtain the complex solutions at different mixing ratios (OS/ β-lg (*w*/*w*): 1:10, 2:10, 3:10, 5:10). The flow rate of OS and β-lg dispersions were 0.5 mL/min and 2 mL/min, respectively. The total concentration of the mixtures was 1.5% (*w*/*v*). The pH of the mixtures was set at the desired value (2.0–5.2) using HCl (0.1 M) and NaOH (0.1 M). Sodium azide (0.02%, *w*/*w*) was added to prevent bacterial growth. Then, the complex solutions were kept at room temperature for 24 h to form a coacervate phase equilibrium. The coacervate phase of the mixture was separated for subsequent measurements. The turbidity of the samples at 600 nm was measured using a UV-visible spectrophotometer (Ultrospec 2000, Pharmacia Biotech, Uppsala, Sweden) at room temperature. The critical pH values (pHc, pHφ1, pHφ2, pHopt) were determined according to the previous literature [18]. The particle size distribution, mean size, and zeta potential of all the samples were measured using Malvern Nano-ZS ZEN3600 instrument (Malvern Panalytical, Malvern, UK). Each sample was tested in triplicate at 25 °C with a backscattered angle of 173°. The contact angle and interfacial tension of OS/β-lg complex particles between aqueous and oil phases were measured (for more details, see Supplementary Materials, Section 1.12). The samples from each group were assayed in triplicate, and their detailed information on the reaction conditions and composition are shown in Table 1.

### 2.4. Preparation of OS/β-lg Particle-Stabilized Emulsions

The collected OS/β-lg complex coacervates (OS-0.72, pH = 3.6, OS/β-lg mixing ratio of 2:10) were diluted for emulsion preparation (1–6%). Oil-in-water emulsions (15 mL) were prepared at the desired oil/water ratio (1:20, 1:10, 1:4, and 1:1, *v*/*v*) and NaCl strengths (0, 20, 100, 200 mmol/L) using the medium-chain triglyceride (MCT) oil. Specifically, the MCT oil was added, and homogenization was performed at 9000 rpm for 3 min. The mean droplet size (Malvern Masterszier 3000), microrheological behavior, and visual interface structure of the obtained emulsions were observed (for more details, see Supplementary Materials, Section 1.2). Moreover, the certified astaxanthin (AST) standard was dissolved in MCT oil (0.1%, *w*/*w*) to prepare AST-enriched emulsions at a 1:4 oil/water ratio (3 mL of oil: 12 mL of 4% OS/β-lg particle solution).

### 2.5. Physical and Chemical Stability of AST-Enriched Emulsions

After preparation of AST-enriched emulsions, they were incubated at different temperatures (30–90 °C) for 12 h. For ultraviolet treatments, the prepared emulsions were irradiated at various time intervals (0–12 h) at 26 °C (48 W, 254 nm). In addition, 40 μL of sodium hypochlorite was dropped into 25 mL beakers containing 20 mL of emulsions. The mixtures were kept in dark at 4 °C for various times (0–120 min). Similarly, the MCT oil phase containing AST (0.1%, *w*/*w*) were processed by the same procedures. The droplet size and AST remaining content of all samples were observed timely.

### 2.6. In Vitro Digestion of AST-Enriched Emulsions and Free Fatty Acid Release

The prepared emulsions were sequentially passed through simulated digestive fluids (SSF: simulated salivary fluid, SGF: simulated gastric fluid, SIF: simulated intestinal fluid), which were exactly prepared as published by Liu et al., with minor modifications [19]. The titration method was employed to measure the release of free fatty acids [20]. The CLSM images of AST-enriched emulsions through gastrointestinal fluids were observed and collected (for more details, see Supplementary Materials, Sections 1.3 and 1.4).

### 2.7. Statistical Analysis 

All the experiments were performed in triplicate, and the results are shown as means ± standard deviation. The one-way analysis of variance (ANOVA) was used to determine significant difference, and the significance level was set to 0.05.

## 3. Results and Discussion

### 3.1. Structure Characterizations of Oxidized Starch and Commercial β-lg

The structural information of the prepared OS is listed in Table S1. The degree of carboxyl substitution (DS) of the three OS samples presented at 0.25, 0.72, and 1.65%. In comparison with native starch, the appearance of the main peak at 1738 cm^−1^ (C=O stretching vibration) demonstrated the successful introduction of the carboxyl moiety (Figure S1). The surface ζ-potential of the OS-0.72 solution reached saturation at ≥2 mg/mL (Figure S2). The absolute potential value of the OS dispersions at 2 mg/mL increased with the increase in DS and pH values and then reached a steady state at pH 5.0 (Figure S3). Moreover, the experimental isoelectric point (pI) of β-lg was in the range of 4.8–5.0 (Figure S4), revealing that the complex coacervation between OS and β-lg occurred at pH ≤ 5.0.

### 3.2. OS/β-lg Complex Coacervation through Microfluidic Platform

**Figure 1:**
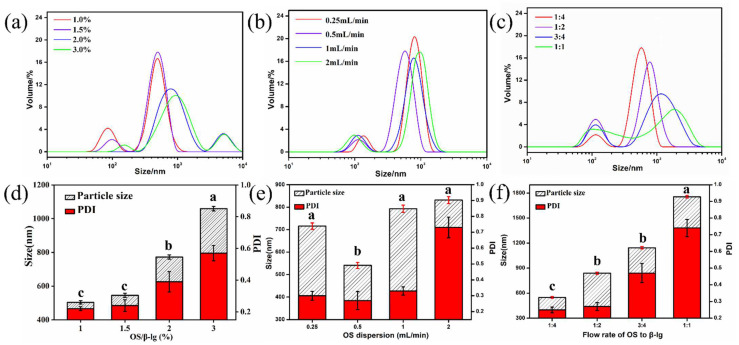
Particle size distribution (**a**–**c**), particle size, and PDI (**d**–**f**) of OS/β-lg complex particles at different total concentrations, flow rates of pump 1, and flow rate ratios of pump 1 to 2. Bars with different letter superscripts are significantly different at *p* < 0.05.

The driving forces for the formation of polysaccharide/protein complex particles in the microfluidic chip mainly are large fluid shear forces and wall interaction forces, which are significantly associated with the dispersion concentration and flow rate of all pumps [21]. Herein, the effect of the OS/β-lg total concentration, flow rate of pump 1, and flow rate ratio of pump 1 to 2 on the particle size distribution and PDI of OS/β-lg complex particles under constant conditions (OS-M, pH = 3.6, and OS/β-lg ratio of 2:10) is depicted in Figure 1. With the increase in the OS/β-lg total concentration, there is a prompt increase in average particle size and PDI of the OS/β-lg colloidal particles, and the smallest particle size and PDI presented at around 500 nm and 0.22 at 1.0% (*w*/*v*) (Figure 1a,d). This can be ascribed to the enhanced viscosity of the OS/β-lg solution in the microfluidic devices, which suppressed the fluid shear and wall interaction forces of the microfluidic chip. This decrease in the joint interaction forces reduced the fragmentation of the OS/β-lg composites, thus leading to the formation of large-size particles and decreasing the uniformity of the particle size distribution at higher concentrations. Moreover, Figure 1b,e shows a tendency of first decreasing and then increasing in the particle size and PDI with the increase in the flow rate of pump 1 from 0.25 to 2 mL/min under stationary conditions. The minimum particle size and PDI of the OS/β-lg complex were found to be around 540 nm and 0.27 at 0.5 mL/min. This finding can be attributed to the fact that a lower flow rate can prolong the condensation time of OS and β-lg and then result in the aggregation of OS/β-lg complex particles. Similarly, the higher flow rate increased the fluid shear force but shortened their condensation time and weakened the wall interaction forces, thus leading to the limited coacervation of the OS and β-lg molecules and the formation of large-size composites. Meanwhile, the flow rate ratios of two dispersions in the microfluidic chip can also affect the fluid shear force and particle properties of the obtained composites. As shown in Figure 1c,f, the particle size and PDI of the OS/β-lg composites gradually increased as the flow rate difference of pump 1 and 2 decreased. The OS/β-lg coacervates showed the smallest particle size (548.1 nm) and PDI value (0.25) at the flow rate ratio of 1:4. Such results can be attributed to the fact that the larger the flow rate differences in the sample inlet of the microfluidic chip, the stronger the shear forces are on the OS/β-lg coacervates, thereby strengthening the fragmentation and monodisperse degree of the composite particles. In summary, the optimal device parameters for the formation of the OS/β-lg complex particles were found to be 1.5% of the OS/β-lg total concentration, 0.5 mL/min of the flow rate (pump 1), and 1:4 of the flow rate ratio (pump 1 to 2).

### 3.3. Effects of pH, DS, and Mixing Ratio on OS/β-lg Complex Coacervation

**Figure 2:**
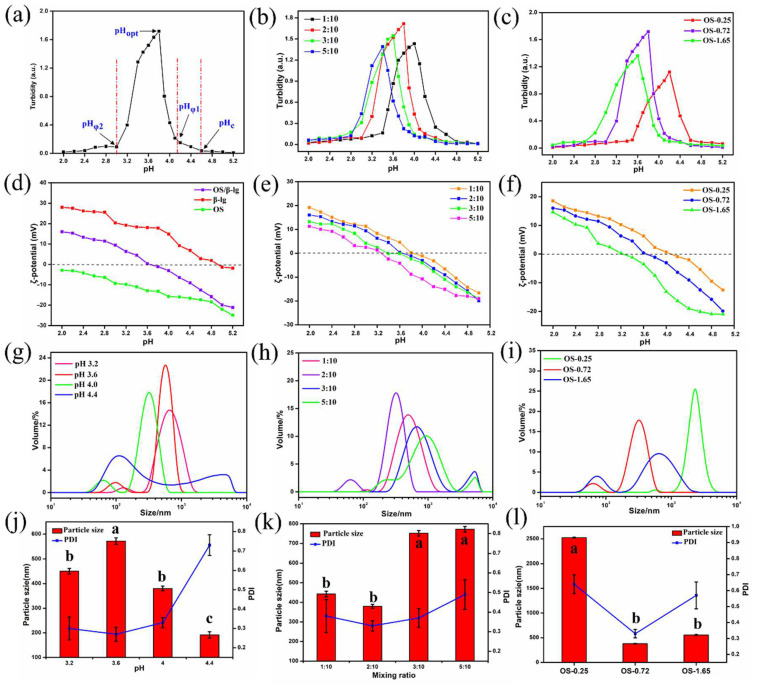
Turbidity curve (**a**–**c**) and zeta potential (**d**–**f**) of OS/β-lg complex dispersions at different pHs and OS/β-lg mixing ratios ((**a**): OS-0.72, OS/β-lg mixing ratio of 2:10; (**b**): OS-0.72; (**c**): OS/β-lg mixing ratio of 2:10); and particle size distribution (**g**–**i**), average size, and PDI (**j**–**l**) of OS/β-lg complex coacervates at different pHs, DSs, and OS/β-lg mixing ratios. Bars with different letter superscripts are significantly different at *p* < 0.05.

The pH and mixing ratio of biopolymers are important factors affecting the complex coacervation of protein–polysaccharide. The pH controls the ionization degree of functional groups and the electrostatic interaction strength. The mixing ratio handles the electrical charge balance and electrostatic interaction-based biopolymer complex formation [22]. Figure 2a–f illustrates the effects of the pH, mixing ratio of OS to β-lg, and DS of oxidized starch on turbidity, critical pH values (pHc, pHφ1, pHφ2, pHopt), and ζ-potential of the OS/β-lg complex coacervates at the total concentration of 1.5% (*w*/*v*). The highest interaction (OS-0.72) was observed at the mixing ratio of 2:10 and pH 3.7. The carboxyl groups in the polysaccharides interact with the amino groups in protein to form an OS/β-lg complex at an acidic pH. When the mixing ratio of OS/β-lg was more than 2:10, the complexation was refrained due to the excess amount of OS. Meanwhile, when the ratio was less than 2:10, the maximum turbidity decreased due to the preference of the β-lg molecules to interact with themselves rather than the polysaccharide. Interestingly, the optical density of the OS/β-lg complex decreased at the mixing ratio of 2:10 when using OS-1.65. As the reaction sites of the amino groups in β-lg were limited, the amount of carboxyl groups in OS-1.65 was too high for the complex formation. This phenomenon is consistent with the reported findings on whey protein isolate and flaxseed gum complexation [23].

**Figure 3:**
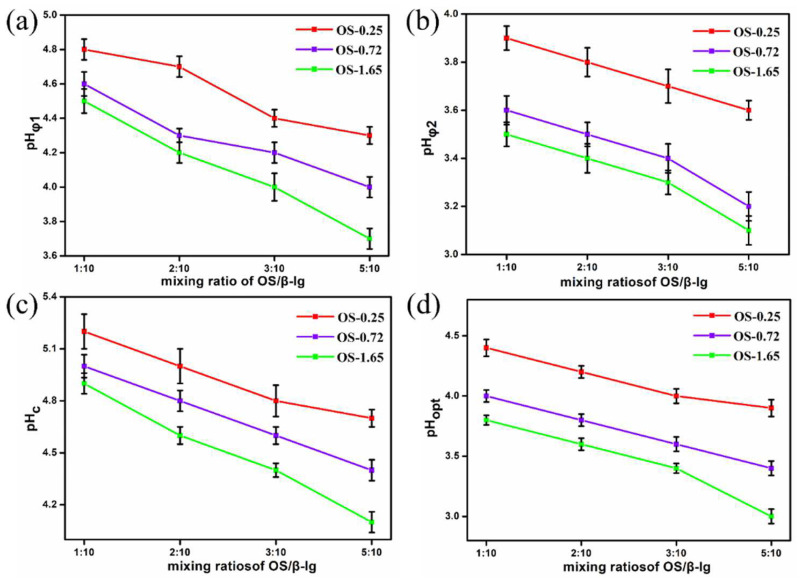
The changes in pHφ1 (**a**), pHφ2 (**b**), pHc (**c**), and pHopt (**d**) of OS/β-lg complex dispersions at different DSs and OS/β-lg mixing ratios.

In the turbidity–pH diagram of the OS/β-lg complex coacervates at the highest optical density, four different phases were observed (Figure 2a). When the pH > pH_c_, there was no protein–polysaccharide interaction as a result of coulombic forces [18]. When pH_φ1_ < pH < pH_c_, the slight increase in turbidity revealed the formation of a soluble OS/β-lg complex with a certain amount of negative net charges [24]. When pH_φ2_ < pH < pH_φ1_, the insoluble OS/β-lg complex appeared by decreasing the pH and presented a steep increase in turbidity until at pH 3.7 (pH_opt_), suggesting the maximum points of electrostatic OS-β-lg interaction for the insoluble OS/β-lg complex formation. Then, the dissociation of the OS/β-lg coacervates started due to the protonation of the carboxyl groups in the OS until complete dissociation at pH_φ2_ [25]. Once the pH was below this point, contributive electrostatic interaction between the OS/β-lg mixtures gradually diminished. Furthermore, the four critical pH points all shifted to lower values as the mixing ratio of OS/β-lg increased from 1:10 to 5:10, (Figure 2k and Figure 3). Similar results on pea protein isolate–gum Arabic as well as canola protein isolate–alginate complex have been reported [18,26].

Figure 2d demonstrates the surface potential of OS, β-lg and their complex coacervates (OS-0.72, mixing ratio of 2:10, pH 3.6). At pH < 5.5, the amine and carboxyl groups of β-lg became protonated, and the protein surface charge was positive. The anionic OS had negative charge at all pH values, but the amount of the charge depended on the degree of carboxyl substitution on the OS. At pH 3.6, an electrostatic protein–polysaccharide interaction happened and formed the OS/β-lg complex without net surface charge. This value was equal to pHopt, suggesting that the electrostatic interaction was the main driving force in the stability of the OS/β-lg complex [27]. This finding is agreement with the work by Anal et al., who reported that pH had a direct effect on the ζ-potential of complex solutions between chitosan and casein [28]. In summary, the optimal coacervation condition for the OS/β-lg complex particles presented at the mixing ratio of 2:10, OS-0.72, and pH 3.6.

### 3.4. Particulate Properties of OS/β-lg Complex Coacervates

The particulate properties of complex coacervates such as particle size and wettability are extremely fundamental to its performance as Pickering stabilizers. Figure 2g–l demonstrates the effect of the pH, mixing ratio, and DS of oxidized starch on the particle size distribution of OS/β-lg composites. The particle size of the OS/β-lg particles presented a tendency to increase first and then decrease as the pH decreased, finally reaching 571.8 nm at pH 3.6. Moreover, the particle size and PDI of the OS/β-lg particles first decreased and then increased as the mixing ratio of OS/β-lg and DS of oxidized starch increased. These results are agreement with the changes in turbidity of the OS/β-lg complex dispersions at different pH conditions, namely, the strongest electrostatic interaction, and the most uniform particles were observed at pH3.6, with an OS/β-lg mixing ratio of 2:10, and 0.72% of DS for oxidized starch. When the mixing ratio was less than 2:10, the protein molecules preferred to interact with themselves, forming a certain number of β-lg-β-lg aggregates.

The current understanding holds that the wetting properties of the protein–polysaccharide complex are mainly affected by the pH, mixing ratio, and the type of polysaccharides. Colloidal particles can endow Pickering emulsion with great stability when its three-phase contact angle is close to 90° [29]. As shown in Table 2, the three-phase contact angle of the OS/β-lg complex coacervates tended to first increase and then decrease with the decrease in pH. When the DS of oxidized starch reached 0.25% and 1.65%, the binary composites presented excess β-lg-caused hydrophobicity and excess OS-caused hydrophilicity. The increased DS and OS/β-lg mixing ratio contributed to the hydrophilicity of the prepared OS/β-lg particles due to the increasing carboxyl groups. As expected, the obtained OS/β-lg complex at the mixing ratio of 2:10 showed a contact angle of 86.68° at pH 3.6. According to the definition of desorption energy [29], θ_o/w_ ≈ 90° revealed that complex particles need maximum desorption energy from the oil/water interface. Therefore, the optimal OS/β-lg complex coacervates can tightly assemble at the oil–water interface, which can encapsulate the emulsion droplets for preventing self-aggregation. Correspondingly, the OS/β-lg complex-formed emulsion droplets reached a minimum interface intention (4.84 mN/m) at pH 3.6 (Table 3), which was in agreement with the contact angle measurements. Considering that ΔH = −21.4 kcal/mol and −TΔS = 14.4 kcal/mol were attributable to the binding of OS-0.72 to the β-lg at the mixing ratio of 2:10 (pH 3.6), this binding reaction was partially driven by hydrogen binding with a higher KD value of 7.08 × 10^−6^ (Figure S5). These results demonstrate that OS-0.72 and β-lg voluntarily condensed in the microfluidic channels through electrostatic interaction and hydrogen bonding. So, the complex particles formed at optimal conditions (a mixing ratio of 2:10, pH 3.6, OS-0.72) were selected to perform the following emulsion preparation.

### 3.5. Emulsifying Performance of OS/β-lg Complex Particles

#### 3.5.1. Droplet Size and Microscopic Morphology

**Figure 4:**
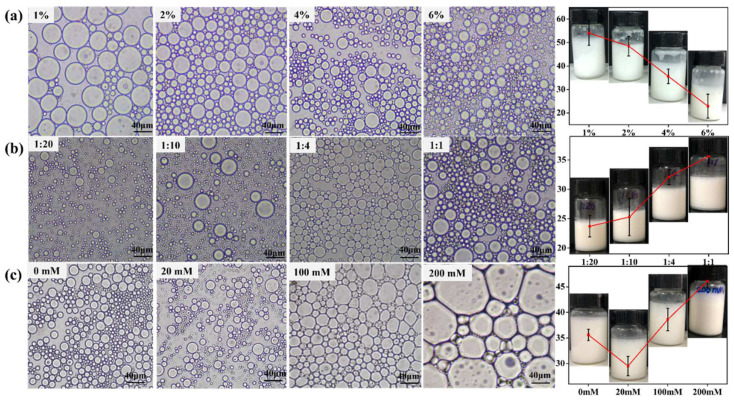
The droplet size (μm) and visual and micromorphologies of OS/β-lg complex stabilized emulsions at different solid concentrations (**a**), oil/water ratios (**b**), and ionic strengths (**c**). Scale bars are 40 μm.

Droplet size and the optical microscopy photographs of OS/β-lg complex-stabilized emulsions were observed at different oil/water ratios, solid concentrations, and ionic strengths (Figure 4). Even a particle concentration of 1% could fabricate stable Pickering emulsions, thus showing the superior emulsifying properties of the prepared OS/β-lg complex at the optimal condition. When the particle concentration increased from 1 to 6%, the droplet size shifted from 54.1 to 22.9 μm. Adequate colloidal particles can be closely adsorbed on the oil/water interface to form a thick and compact film, which suppressed the probability of coalescence after the collision of droplets [30]. In addition, the droplet size showed a tendency of increasing as the oil fraction increased. The obtained emulsions began to slightly separate into oil and water phases at the oil/water ratio of 1:1. The higher oil contents resulted as the emulsified colloidal particles were not sufficient to stabilize the emulsions, resulting in the presence of droplet aggregates and Ostwald ripening [6]. Moreover, the droplet size decreased from 35.6 to 29.5 μm with a NaCl strength of 20 mmol/L. This phenomenon can be explained by the fact that the improved hydrophobicity of the OS/β-lg complex increases its adsorption on the oil/water interface and then reduces the emulsion size. The further increased salt levels resulted in the gradual increase in droplet size up to demulsification. This can be attributed to the fact that the excess of NaCl enhances the electrostatic repulsion of OS/β-lg particles, thus leading to the decrease in adsorbed particles and the larger droplet size. These results are supported by the report of Li et al., who revealed that the higher NaCl concentration results in the droplet coalescence of Pickering emulsions stabilized by chitosan hydrochloride/carboxymethyl starch complex particles [31].

#### 3.5.2. Microrheological Properties

**Figure 5:**
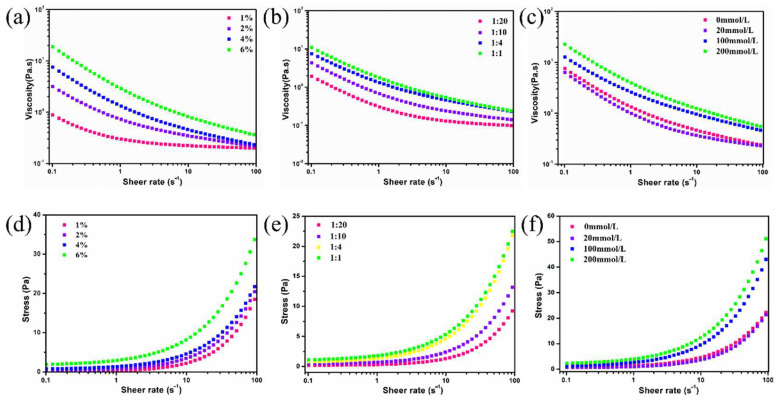
Viscosity (**a**–**c**) and shear stress (**d**–**f**) of OS/β-lg complex stabilized emulsions at different particle concentrations, oil/water ratios, and ionic strengths.

Steady stress rheological measurements were performed to investigate the microrheological properties of the obtained emulsions. As shown in Figure 5a–c, the apparent viscosity of all emulsions gradually decreased in the shear rate range of 0.1 to 100 s^−1^, showing typical non-Newtonian shear-thinning behavior. This phenomenon is associated with the disaggregation effect of emulsion droplets subject to force field, which was also observed by Dai et al. [32]. In comparison, the increased particle concentration, oil phase fraction, and ionic strengths contributed to the increase in emulsion apparent viscosity, which was in accordance with the microrheological constants of soy glycinin-stabilized Pickering emulsions [33]. The higher particle contents increased the emulsion viscosity via its thickening effect and thus prevented the aggregation of droplets for emulsion stability.

Figure 5d–f shows the shear stress variations of the prepared emulsions against shear rates. The relationship between the shear stress and the shear rates was non-linear, while the obtained emulsions presented the properties of pseudoplastic fluids. Then, Table S2 lists the apparent parameters via the fitting of rheology data using the Herschel–Bulkley model (R^2^ > 0.99). The lower yield stress suggested the emulsions tended to phase separation, while the$\;\tau_{0}$ and κ values gradually increased with the increase in particle amount and oil fraction, and the n value correspondingly decreased. These findings suggested the formation of a more compact 3D gel network structure with the increase in OS/β-lg particles. However, its apparent viscosity and yield stress tended to decrease first and then increase with the increase in NaCl addition. This may be ascribed to the fact that the electrostatic screening of Na^+^ partially suppressed the electrostatic protein–polysaccharide interaction and disrupted the network structure of emulsions [34]. The suffered viscous resistance of the emulsions decreased the setting rate of the emulsion droplets and further enhanced the global stabilization of the emulsion-based delivery systems.

#### 3.5.3. Visualization of Interfacial Structure

**Figure 6:**
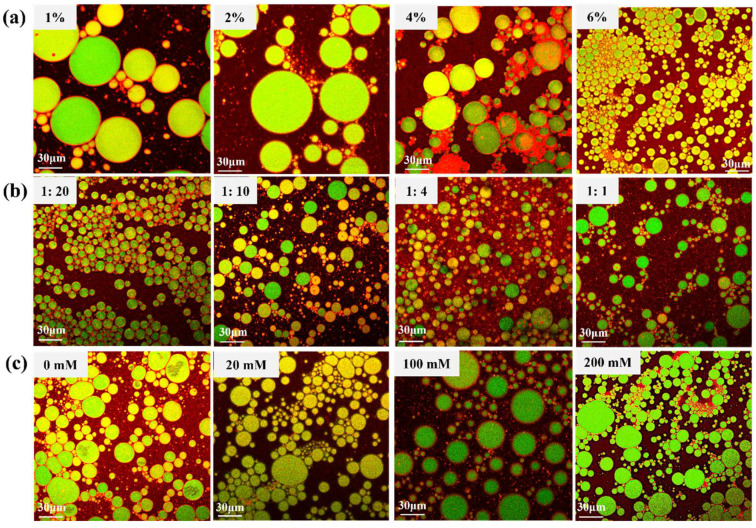
CLSM images of OS/β-lg complex stabilized emulsions at different particle concentrations (**a**), oil/water fractions (**b**), and ionic strengths (**c**). Scale bars are 30 μm.

CLSM images of the dyed emulsion droplets at different particle concentrations, oil-to-water ratios, and ionic strengths are shown in Figure 6. Therein, MCT oil showed in green, the OS/β-lg complex in red, and the emulsion droplets in yellow. The red fluorescence was visualized in the gap of droplets, while the green fluorescence was clearly observed in the internal of droplets, confirming the formation of oil-in-water emulsions. This interfacial structure revealed that the anchoring of the OS/β-lg complex at the oil/water interface resulted in the formation of a compact interface layer encircling the ball-shaped oil droplets, which can protect the droplets against coalescence and Ostwald ripening. With the continued addition of the OS/β-lg complex, the droplet size dramatically decreased in accordance with the DLS results. When the addition amount reached more than 4%, the red fluorescence occupied the free space of the droplet gaps. These findings indicated that the excess OS/β-lg complex involved reactions such as cross-linking and thickening with the self-assembled droplets to enhance the network structure of the obtained emulsions [29]. Meanwhile, the droplet size gradually increased as the oil fractions increased due to the lack of insufficient solid particles to emulsify the extra oil phase (Figure 6b). The solid packing density can directly affect the droplet size of emulsions as well as the apparent viscosity [35]. However, there was no clear-cut phase separation even at an oil volume fraction as high as 50%, suggesting the attractive potential of the prepared OS/β-lg complex to be used for high internal phase emulsions. In addition, the droplet size revealed a trend of first decreasing and then increasing with the increase in Na^+^ strength. At 200 mmol/L, the oil droplets began to coalesce and slight phase separation commenced, indicating that extensive droplet aggregation occurred at high ionic strengths [35].

#### 3.5.4. Physical and Chemical Stability of AST-Enriched Emulsions

**Figure 7:**
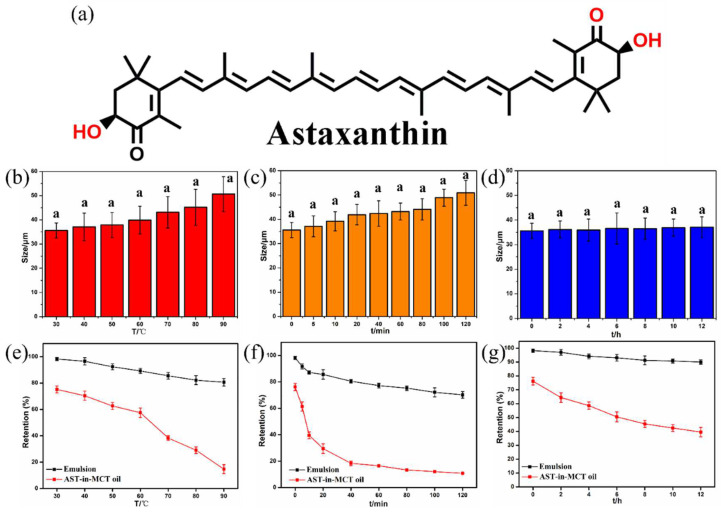
Chemical structure of AST (**a**). Changes in droplets size and AST retention amount of OS/β-lg complex stabilized emulsions facing thermal treatment (**b**,**e**), oxidative stress (**c**,**f**), and ultraviolet irradiation (**d**,**g**). Bars with different letter superscripts are significantly different at *p* < 0.05.

There is a limited application of AST (Figure 7a) in various food formulations as it is extremely susceptible to chemical degradation when exposed to oxygen, light, high temperatures, and extreme pH conditions [36]. Here, the AST-loaded emulsions were prepared at the oil–water ratio of 1:4 and 4% of particle concentration. The encapsulation efficiency and droplet size of the obtained emulsions presented at 98.2%(Figure S6) and 35.6 μm (PDI = 0.29), respectively. Figure 7 illustrates the effect of thermal treatment, oxidative stress and ultraviolet irradiation on droplet size and AST retention of the OS/β-lg complex stabilized emulsions. With the increase in heating temperature from 30 to 90 °C, the droplet size of emulsions slightly increased from 35.6 to 50.6 μm, and the AST retention content showed a minor decrease from 98.25% to 80.62% (Figure 7b,e). This finding was in agreement with the changes in droplet size of AST-loaded emulsions stabilized by chitosan/pectin particles [37]. Interestingly, the AST amount shifted to 70.21% after 2 h of strong oxidative stress, while its droplet size increased up to 50.9 μm (Figure 7c,f). This may be due to the fact that the electrostatic OS-β-lg interaction was affected by the ionization of -ClO^−^ [38]. No significant changes in the droplet size and AST retention were observed after ultraviolet irradiation for 12 h, demonstrating the excellent stability of the emulsions against long-time exposure to ultraviolet irradiation. This was supported by the previous report of Xiao et al., who revealed that the additional protection effects through the physical layers of kafirin particles can shelter the captured curcumin from direct ultraviolet radiation [39]. In comparison, the remarkable chemical degradation of AST dissolved in MCT oil was observed when exposed to the corresponding process conditions (Figure 7e–g).

### 3.6. Gastrointestinal Fate of AST-Enriched Emulsions

**Figure 8:**
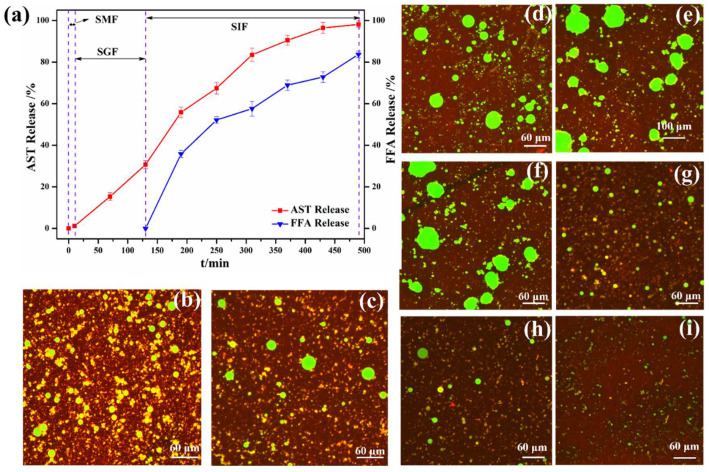
Release behaviors of AST-enriched emulsions in simulated digestion fluids (**a**). Fluorescence micrographs of AST-enriched emulsions after in vitro digestion ((**b**): SMF at 10 min; (**c**): SGF at 2 h; and (**d**–**i**): SIF at 1, 2, 3, 4, 5, and 6 h). Scale bars are 60 μm.

The release performance and bioavailability of AST captured in emulsions appears to be highly instructive for the construction of food-grade emulsions. As shown in Figure 8a, the digestibility of AST was less than 1% after co-incubation with simulated saliva for 10 min, suggesting that the emulsion has little oral collapse [40]. When the processed emulsions were transferred from mouth to the SGFs, the captured AST was quickly released in the first 2 h, which might be due to the liberation of the AST embedded in the interfacial layers of oil droplets. A certain resistance of emulsions to pepsin might be ascribed to that the hindrance effect of high-viscosity emulsion structures on the diffusion of pepsin hydrolysates [41]. Finally, when the emulsions were incubated with the SIFs, the cumulative release of AST significantly increased and reached up to 98.12%. This can be attributed to the droplet breakdown of the AST-enriched emulsions from pancreatin enzymolysis and bile salts in SIFs, facilitating the hydrolysis of the oil phase and fat-soluble substances [42]. Meanwhile, the release of fatty acid in the intestinal phase gradually increased in the first 120 min and reached up to 83.69% after 6 h, showing the high bioavailability of astaxanthin [43].

To further visualize the changes in the interfacial structure of the prepared emulsions throughout the digestion process, Figure 8b–i depicts the fluorescence images of AST-enriched emulsions under the processing of three simulated digestion phases. A large number of yellow emulsion droplets were still remarkably observed after incubation with simulated saliva for 10 min. When the emulsion was transferred from the mouth to the gastric phase, the formation of bigger-sized, green oil droplets commenced, and its aggregates began to appear in the microscopic fields. Then, when the emulsions passed to the simulated intestine phase, the number and size of green droplets gradually increased within 3 h. There was only a small number of stable droplets after 4 h, until hardly any oil droplets appeared in the view of the micrographs. These findings agreed with the release performance of AST and free fatty acid. The influence of pH and pancreatin on emulsifiers resulted in the disintegration of the OS/β-lg complex particles, thereby improving the transfer of AST into the enterocytes via the micellization of the AST, oil phase, and the OS/β-lg fragment [20].

## 4. Conclusions

In summary, we prepared complex coacervates using oxidized starch and β-lactoglobulin via a dual-channel microfluidic chip. The highest electrostatic complex formation occurred at a pH of 3.6, OS/β-lg mixing ratio of 2:10, and DS of 0.72%. The complex colloidal particles showed a neutral contact angle of 86.68°. The OS/β-lg complex particles formed a densely packed layer on the interface of the oil droplets. Together with the thickening and cross-linking effect from the nonadsorbed particles, this compact layer provided a stable physical barrier to prevent the aggregation of the emulsion droplets. The corresponding astaxanthin-enriched emulsions exhibited the controlled-release pattern, also with excellent storage stability against the extreme treatments. It can be concluded that the prepared emulsions can retard the release of astaxanthin in the gastric phase and improve its diffusion and solubility during intestinal digestion, which can provide the body with a continuous supply of astaxanthin.

## Figures and Tables

**Table 1:** The detailed information regarding the reaction conditions and composition of all prepared OS/β-lg complex particles and the corresponding emulsions.

Particle Samples						Emulsions (MCT Oil Containing 0.1% AST, *w*/*w*)		
Usage (%)	Flow Rate (mL/min)	Flow Ratio	pH	DS (%)	Mixing Ratio	Usage (%)	Oil/Water Ratio	Ionic Strength (mM)
1.0	0.50	1:4	3.2	0.72	2:10	1	1:4	0
1.5	0.50	1:4	3.6	0.72	2:10	2	1:4	0
2.0	0.50	1:4	4.0	0.72	2:10	4	1:4	0
3.0	0.50	1:4	4.4	0.72	2:10	6	1:4	0
1.5	0.25	1:4	3.6	0.25	2:10	4	1:20	0
1.5	0.50	1:4	3.6	0.72	2:10	4	1:10	0
1.5	1.0	1:4				4	1:4	0
1.5	2.0	1:4	3.6	1.65	2:10	4	1:1	0
1.5	0.50	1:4	3.6	0.72	1:10	4	1:4	0
1.5	0.50	1:2	3.6	0.72	2:10	4	1:4	20
1.5	0.50	3:4	3.6	0.72	3:10	4	1:4	100
1.5	0.50	1:1	3.6	0.72	5:10	4	1:4	200

**Table 2:** Contact angle micrographs of OS/β-lg complex coacervates obtained at different pHs, mixing ratios, and DSs of oxidized starch.

pH	Images	CA (°)	Starch	Images	CA (°)	Mixing Ratio	Images	CA (°)
4.4	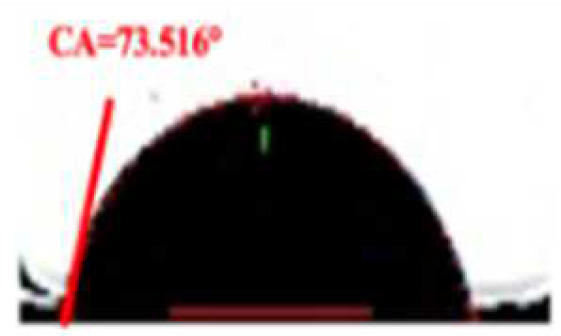	73.52	OS-0.25	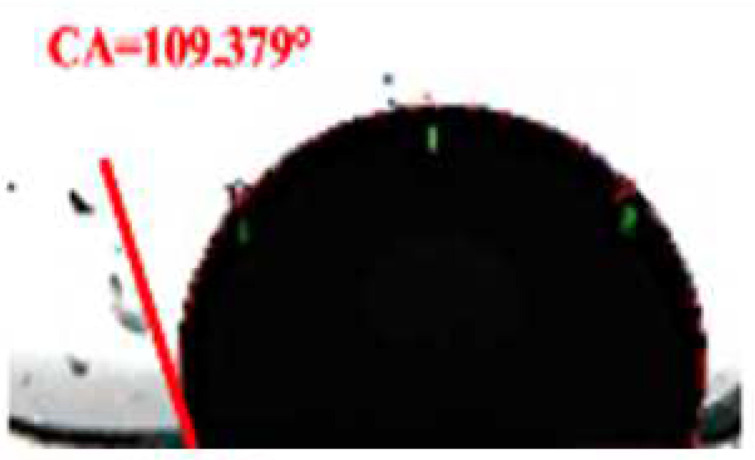	109.38	1:10	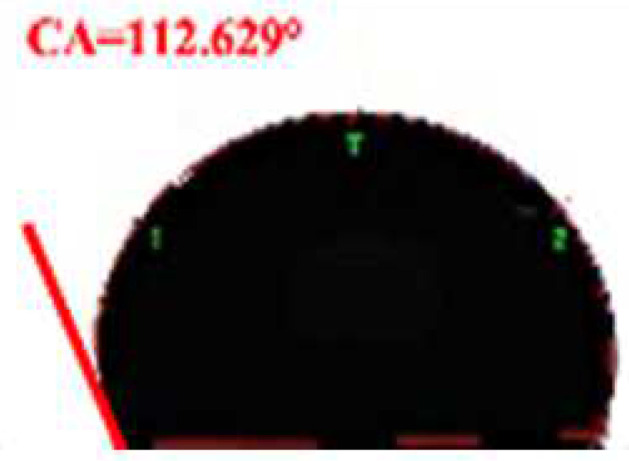	112.63
4.0	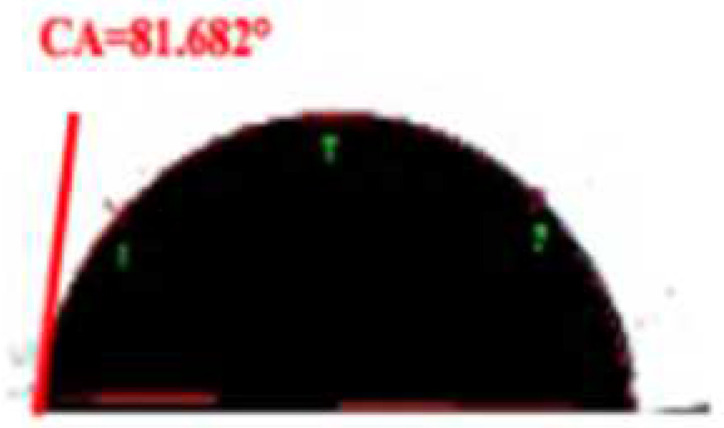	81.68	OS-0.72	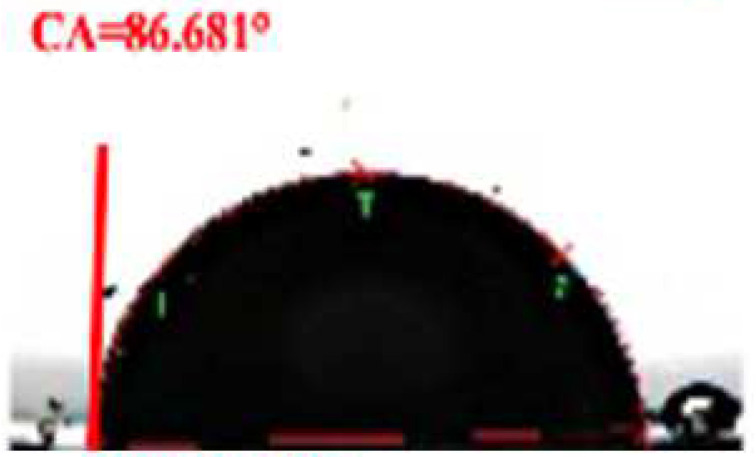	86.68	2:10	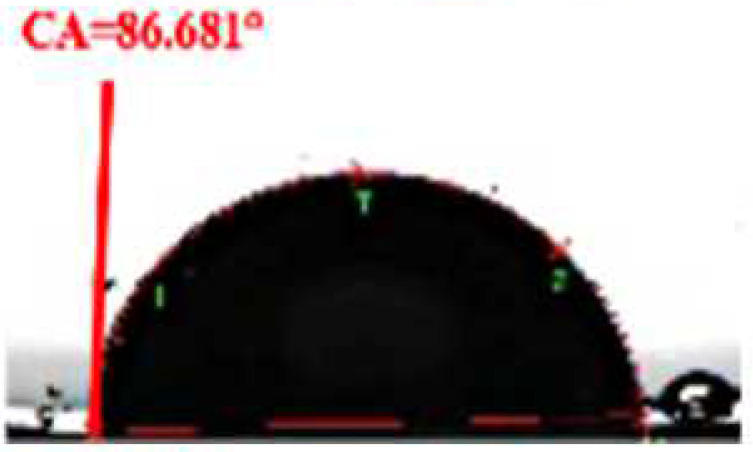	86.68
3.6	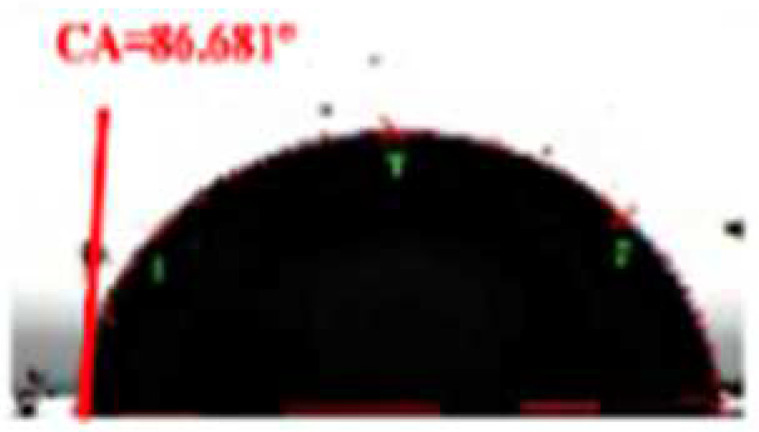	86.68	OS-1.65	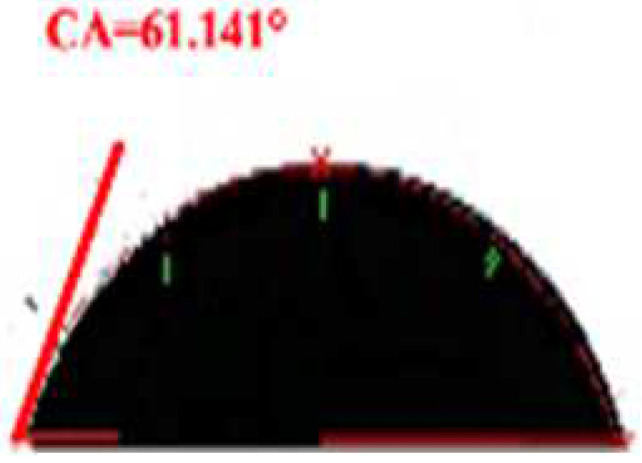	61.14	3:10	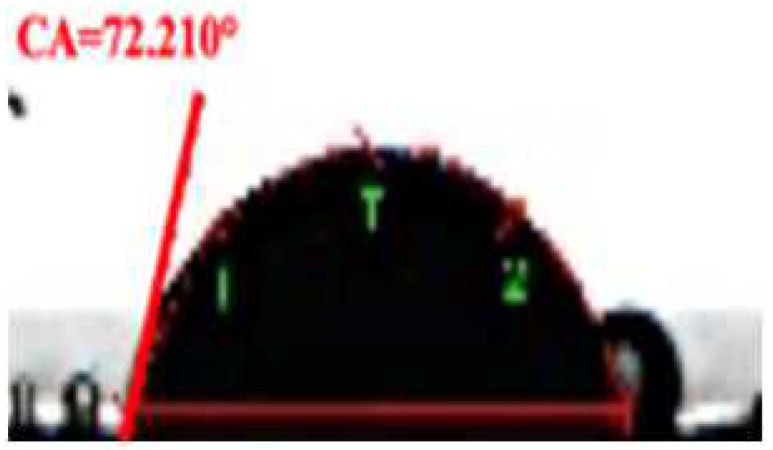	81.26
3.2	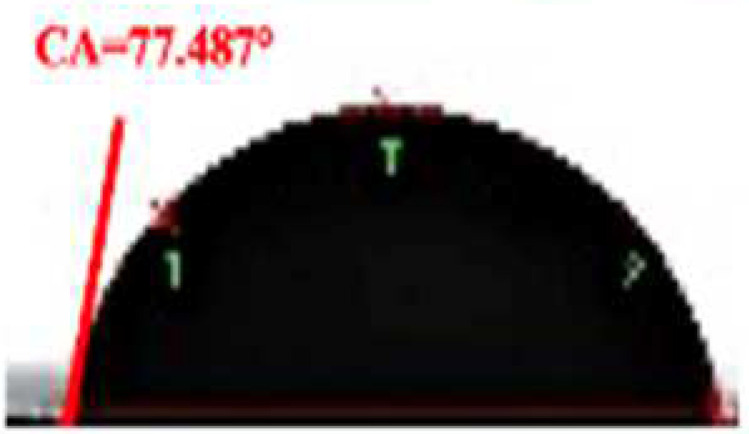	77.49	/	/	/	5:10	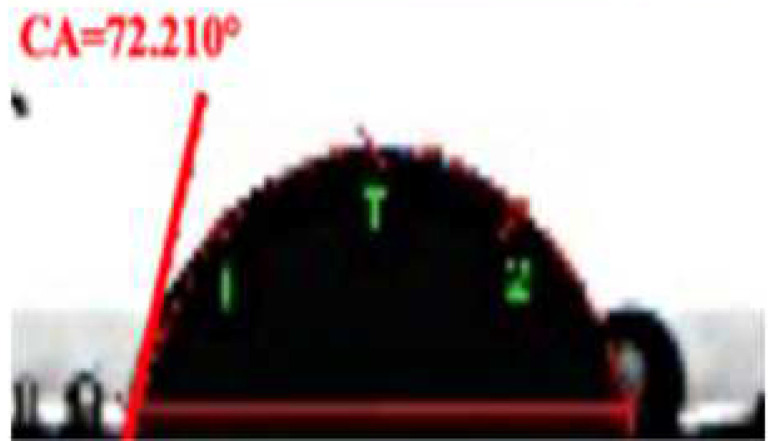	72.21

**Table 3:** Interfacial tension of OS/β-lg complex particles obtained at different pHs, mixing ratios, and DSs of oxidized starch.

pH	Interfacial Tension (mN/m)	OS	Interfacial Tension (mN/m)	Mixing Ratio	Interfacial Tension (mN/m)
4.4	7.18 ^b^	OS-A	10.16 ^b^	1:10	6.55 ^c^
4.0	6.66 ^c^	OS-B	4.84 ^c^	2:10	4.84 ^d^
3.6	4.84 ^d^	OS-C	18.97 ^a^	3:10	10.14 ^b^
3.2	14.11 ^a^	/	/	5:10	17.03 ^a^

^a,b,c,d^ Letters within each row with different superscripts are significantly different at *p* < 0.05.

## Data Availability

The data presented in this study are available on request from the corresponding author. The data are not publicly available duo to privacy restriction.

## Supplementary Materials

The following supporting information can be downloaded at https://www.mdpi.com/article/10.3390/foods11193078/s1: Supporting materials and methods; Table S1: Degree of substitution (DS), molecular weight, RMS radius, and conformation of oxidized starch; Table S2: Apparent constants of the fitting curve based on the rheological data from OS/β-lg complex stabilized emulsions at different particle total concentrations, oil/water ratios, and NaCl concentrations (Herschel–Bulkley model); Figure S1: FT-IR spectra of native starch and oxidized starch; Figure S2: Zeta-potential of OS-0.72 at different concentrations. Bars with different letter superscripts are significantly different at *p* < 0.05; Figure S3: Zeta-potential of native starch and OS at different pH conditions; Figure S4: Zeta-potential of β-lg at different pH conditions; Figure S5: Isothermal titration calorimetric determination on the binding of OS-0.72 to β-lg at the mixing ratio of 2:10 (pH 3.6); and Figure S6: Calibration curve of astaxanthin (0.1% *w*/*v*) in MCT oil phase.
