# Supplementary material for: Preparation of Oxidized Starch/β-Lactoglobulin Complex Particles Using Microfluidic Chip for the Stabilization of Astaxanthin Emulsion
Source: Foods. 2022 Oct 4;11(19):3078. doi: 10.3390/foods11193078 (PMC9563734; doi:10.3390/foods11193078)
Supplement: Supplementary file 1 [file foods-11-03078-s001.zip › foods-1938001-supplementary.pdf]

Supporting Information

Preparation of Oxidized Starch/β-lactoglobulin Complex Particles Using Microfluidic Chip for the Stabilization of Astaxanthin Emulsion

1. Methods

1.1. Contact Angle and Interface Tension

The contact angle and interfacial tension of OS/β-lg complex particles (15 mg/mL) between aqueous and oil phases were measured through an optical contact angle and interface tension measuring instrument (ZJ-7000, Shenzhen, China). In the experiment, the freeze-dried particles were pressed into 1 cm × 1 cm thin square sheets, which was immersed in square tanks containing MCT at room temperature. Then, a drop pf deionized water was softly dropped on the surface of the sheets using a standard micro-syringe. After equilibrium for 30 s, the droplet images were captured by a high-speed camera, and the contact angle was calculated by Laplace-Young equation. For interface intension, 10 mL of OS/β-lg complex particles was transferred into a plastic syringe equipped with a stainless-steel needle prior to submerge in an MCT bath to a 5-mm depth. Then, samples were slowly injected to generate droplets at the needle tip. After balance in 20 min, the droplet images were captured by a high-speed camera and the profile data were automatically calculated in the term of interface intentions.

1.2. Observation of Microrheological Behavior and Interface Structure

The visual morphology of the prepared emulsions were measured through an optical microscope (Carl Zeiss, Oberkochen, Germany) equipped with a digital camera. Confocal Laser Scanning Microscopy (IX3-CBH, Olympus, Tokyo, Japan) was used for interfacial adsorption observation of the stained droplets before and after in vitro digestion. In the experiment, the emulsion droplets (1 mL) were dyed with a mixed fluorescent staining solution (20 μL) consisting of 0.1 wt% Nile Red for oil phase stain and 0.1 wt% Nile Blue for OS/β-lg composite particles stain. Then, 20 μL of the stained droplets were placed in the groove of microscope slides and fitted with a coverslip for interface structure observation. The used excitation wavelengths were 488 nm for Nile Red and 633 nm for Nile Blue. The scanning was conducted with an acquisition speed of 100 Hz at a resolution of 1024×1024 pixels. The microrheological behavior of astaxanthin emulsions was investigated by a rotational rheometer (Physica MCR 301, Anton Parr, Graz, Austria). Briefly, the dynamic viscosity and shear stress of emulsion samples were measured at 25 °C using a parallel plate (60 mm diameter, 1 mm thickness, gas width of 500 μm). Viscosity (Pa.s) and apparent shear rate (Pa) versus shear rate (S^{-1}) were plotted.

1.3. In vitro Digestion of AST-Enriched Emulsions and Free Fatty Acid Release

For in vitro digestion experiments, 7.5 mL of SSF (pH=6.8) was first mixed with 7.5 mL of the prepared emulsions at 37°C and the mixed solutions was stirred (100 rpm) in a thermostatic water bath at 37°C for 10 min. Then, 15 mL of the solutions after mouth digestion was mixed with 15 mL of SGF (pH=1.2). The mixture was constantly incubated (100 rpm) in water bath for 2h at 37°C. After that, 30 mL of the mixtures after gastric digestion was added to 30 mL of SIF (pH=6.8) and incubated for 6 h at 37°C under a constant shaking of 100 rpm. For each sample, 5mL of the proceed emulsions was added to a 15mL-centrifuge tube, following a centrifugation treatment for 30min (25°C, 1000×g). The oil phase (50μL) was collected and then diluted by the mixture of CH2Cl2 and methanol (2:1, v/v) with a final volume of 5 mL, following a centrifugation treatment for 20 min at 4472×g. The astaxanthin content in the supernatant was determined using

UV/visible spectroscopy at 480 nm. The release rate of AST was calculated by the equation as follows.

$$AST\ Release\ (\%) = 100 \times \frac{\text{AST in oil phase}}{\text{AST in emulsion}} \quad (1)$$

The titration method [1] was employed to measure the release of free fatty acids. Briefly, 5 mL of samples after simulated intestinal digestion was collected and mixed with 10 mL of acetone and 3 drops of 1% w/v phenolphthalein. The mixed solution was titrated with 0.1 M NaOH maintained till the solution gained the pink color. The following equation was employed to determine the released FFA.

$$FFA\ Release\ (\%) = 100 \times \frac{M_{oil} \times c_{NaOH} \times V_{NaOH}\,(\text{t})}{2m_{oil}} \quad (2)$$

Here, Moil is the molecular weight of MCT (g/mol); C_{NaOH} refers to the molarity of NaOH solution (mol/L); V_{NaOH} presents the consumption of NaOH solution at T = t (L); moil means the total usage of MCT in simulated intestinal digestion (g).

2. Supplementary Results

Table S1. Degree of substitution (DS), molecular weight, RMS radius and conformation of oxidized starch.

Starch	DS (%)	Mw (g/mol)	Rg (nm)	Conform. Index	Conformation
OS-A	0.25	2.868×10^{7}	115.3	0.42	random coil
OS-B	0.72	4.317×10^{6}	76.4	0.21	—
OS-C	1.65	7.806×10^{5}	15.5	0.07	—

Table S2. Apparent constants of the fitting curve based on the rheological data from OS/β-lg complex stabilized-emulsions at different particle total concentration, oil/water ratio, and NaCl concertation (Herschel-Bulkley model).

Constants	Particle Concentration (%)				O/W Fraction				NaCl (mM)			
	1	2	4	6	1:20	1:10	1:4	1:1	0	20	100	200
τ_0(Pa)	0.062	0.245	0.644	1.658	0.181	0.392	0.640	0.937	0.648	0.583	0.975	1.874
κ(Pa.s^n)	0.236	0.485	0.693	1.26	0.129	0.293	0.699	0.850	0.698	0.428	1.653	2.130
n	0.96	0.82	0.75	0.71	0.93	0.83	0.75	0.71	0.75	0.85	0.71	0.69
R^2	0.9967	0.9932	0.9968	0.9925	0.9925	0.9986	0.9947	0.9986	0.9907	0.9973	0.9997	0.9918

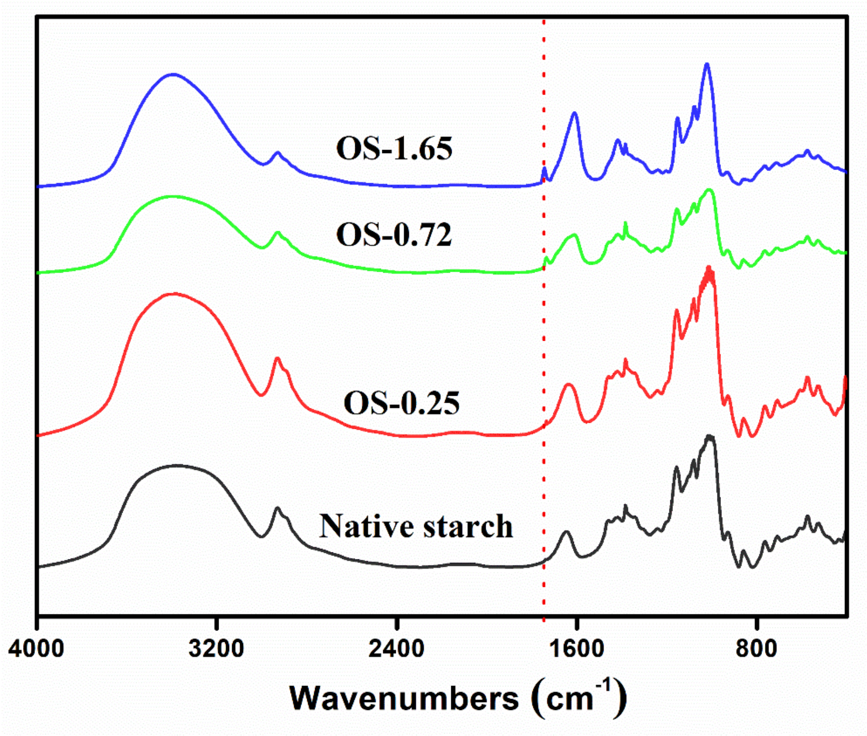

Figure S1. FT-IR spectra of native starch and oxidized starch.

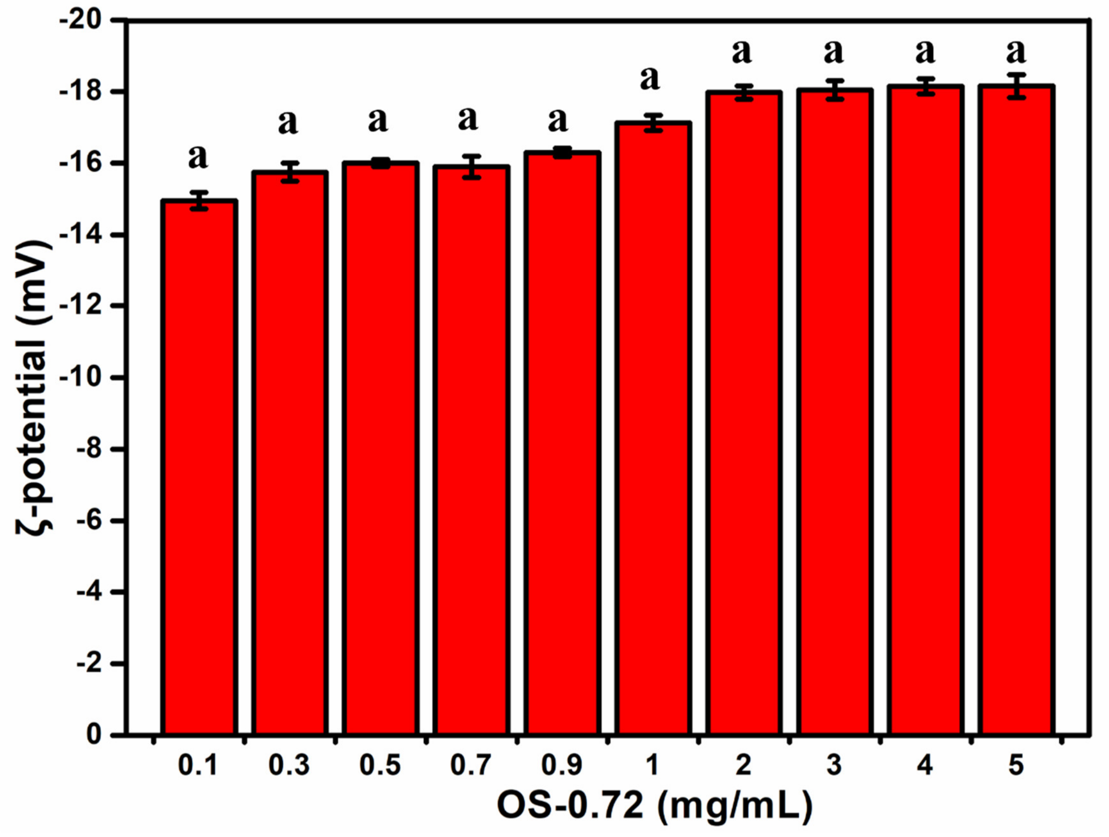

Figure S2. Zeta-potential of OS-0.72 at different concentrations. Bars with different letter superscripts are significantly different at $p < 0.05$.

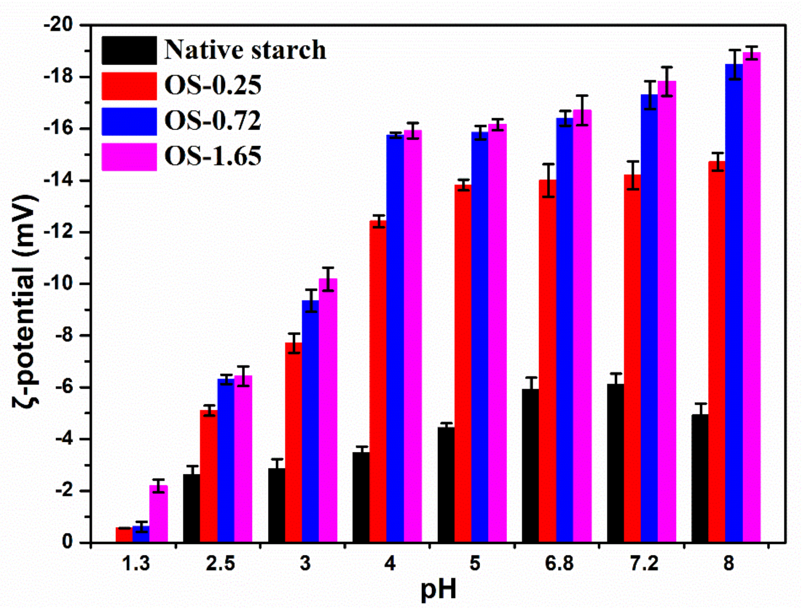

Figure S3. Zeta-potential of native starch and OS at different pH conditions.

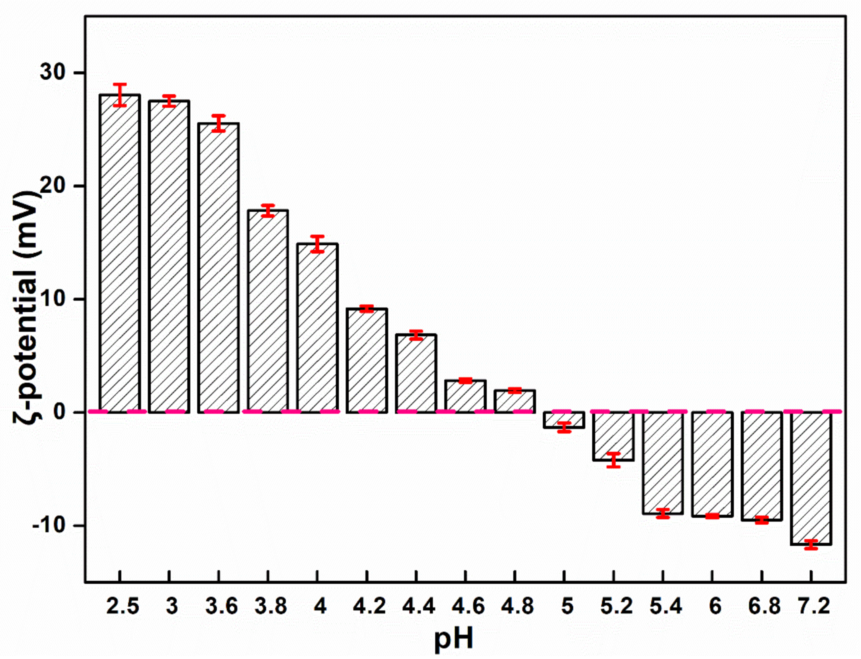

Figure S4. Zeta-potential of β-lg at different pH conditions.

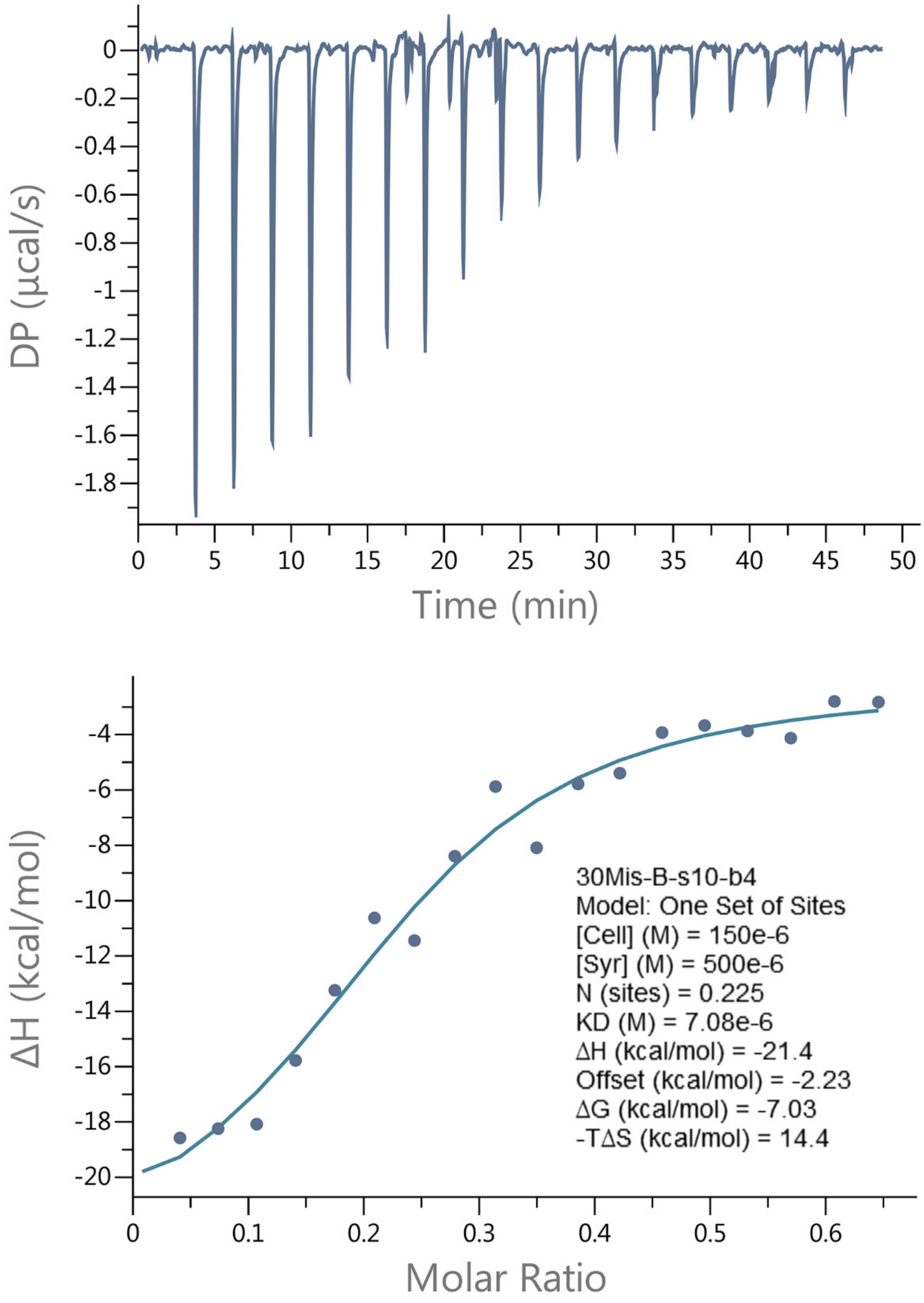

Figure S5. Isothermal titration calorimetric determination on the binding of OS-0.72 to β-lg at the mixing ratio of 2:10 (pH 3.6).

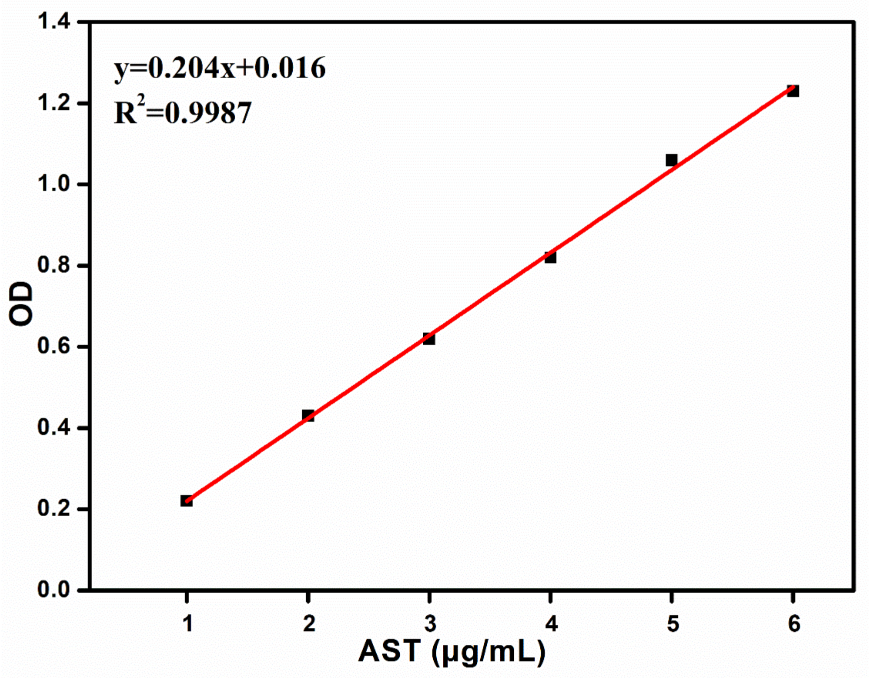

Figure S6. Calibration curve of astaxanthin (0.1% w/v) in MCT oil phase.

Reference

1. Burgos-Díaz, C.; Opazo-Navarrete, M.; Soto-Añual, M.; Leal-Calderón, F.; Bustamante, M. Food-grade Pickering emulsion as a novel astaxanthin encapsulation system for making powder-based products: Evaluation of astaxanthin stability during processing, storage, and its bioaccessibility. *Food Res. Int.* **2020**, *134*, 109244.
